# Evaluation of Retinoids for Induction of the Redundant Gene *ABCD2* as an Alternative Treatment Option in X-Linked Adrenoleukodystrophy

**DOI:** 10.1371/journal.pone.0103742

**Published:** 2014-07-31

**Authors:** Franziska D. Weber, Isabelle Weinhofer, Angelika Einwich, Sonja Forss-Petter, Zahid Muneer, Harald Maier, Willi H. A. Weber, Johannes Berger

**Affiliations:** 1 Center for Brain Research, Medical University of Vienna, Vienna, Austria; 2 Department of Dermatology, Medical University of Vienna, Vienna, Austria; Inserm, France

## Abstract

X-linked adrenoleukodystrophy (X-ALD), the most common peroxisomal disorder, is a clinically heterogeneous disease that can manifest as devastating inflammatory cerebral demyelination (CALD) leading to death of affected males. Currently, the only curative treatment is allogeneic hematopoietic stem cell transplantation (HSCT). However, HSCT is only effective when performed at an early stage because the inflammation may progress for eighteen months after HSCT. Thus, alternative treatment options able to immediately halt the progression are urgently needed. X-ALD is caused by mutations in the *ABCD1* gene, encoding the peroxisomal membrane protein ABCD1, resulting in impaired very long-chain fatty acid metabolism. The related ABCD2 protein is able to functionally compensate for *ABCD1*-deficiency both *in vitro* and *in vivo*. Recently, we demonstrated that of the cell types derived from CD34^+^ stem cells, predominantly monocytes but not lymphocytes are metabolically impaired in X-ALD. As *ABCD2* is virtually not expressed in these cells, we hypothesize that a pharmacological up-regulation of *ABCD2* should compensate metabolically and halt the inflammation in CALD. Retinoids are anti-inflammatory compounds known to act on *ABCD2*. Here, we investigated the capacity of selected retinoids for *ABCD2* induction in human monocytes/macrophages. In THP-1 cells, 13-*cis*-retinoic acid reached the highest, fivefold, increase in ABCD2 expression. To test the efficacy of retinoids *in vivo*, we analyzed ABCD2 mRNA levels in blood cells isolated from acne patients receiving 13-*cis*-retinoic acid therapy. In treated acne patients, ABCD2 mRNA levels were comparable to pre-treatment levels in monocytes and lymphocytes. Nevertheless, when primary monocytes were *in vitro* differentiated into macrophages and treated with 13-*cis*-retinoic acid, we observed a fourfold induction of *ABCD2*. However, the level of ABCD2 induction obtained by retinoids alone is probably not of therapeutic relevance for X-ALD. In conclusion, our results suggest a change in promoter accessibility during macrophage differentiation allowing induction of ABCD2 by retinoids.

## Introduction

X-linked adrenoleukodystrophy (X-ALD; OMIM, phenotype MIM number #300100) is one of the most frequent peroxisomal diseases. The main biochemical characteristic is an impaired metabolism, and thus accumulation, of saturated very long-chain fatty acids (VLCFA) due to mutations in the *ATP-binding cassette (ABC) subfamily D member 1* (*ABCD1*) gene [1]. The encoded peroxisomal ABC transporter ABCD1, (formerly adrenoleukodystrophy protein, ALDP), mediates the trafficking of CoA-activated VLCFA into the peroxisomes for degradation by β-oxidation [2].

X-ALD is a clinically heterogeneous disorder ranging from cerebral inflammatory demyelination (cerebral X-ALD, CALD), leading to death within a few years, to adults remaining presymptomatic for over more than five decades [3]–[5]. The default manifestation of X-ALD is adrenomyeloneuropathy (AMN, mean age-of-onset 28 years), a slowly progressive non-inflammatory axonopathy affecting sensory ascending and motor descending spinal cord tracts [5], [6]. In about one-third of male patients, X-ALD manifests as the rapidly progressive CALD variant occurring independently of AMN. CALD has a typical onset during childhood (35–40%) before the onset of AMN, but develops, less frequently (20%), also in adolescent and adult males [3], [4].

Currently, the only curative treatments for CALD are allogeneic [7], [8] and autologous, genetically corrected, hematopoietic stem cell transplantation (HSCT) [9]. However, these therapies are only reasonable when performed at an early stage, because inflammation still progresses for about 12–18 months after transplantation [9], [10]. Thus, allogeneic and autologous HSCT are not beneficial for CALD patients with an advanced cerebral disease state [7], [8]. Consequently, there is an urgent need for additional therapeutic approaches that are able to immediately halt or slow down the progression of the destructive cerebral inflammation.

Besides ABCD1, two additional ABC half-transporters, ABCD2 and ABCD3, are present in the peroxisomal membrane. Of these, ABCD2 (formerly adrenoleukodystrophy related protein, ALDRP) is the closest homolog sharing 63% amino acid identity with human ABCD1 [11]. Upon overexpression, ABCD2 is able to restore VLCFA metabolism both *in vitro* and *in vivo* [12], [13]. Thus, induction of *ABCD2* gene expression by pharmacological means has been proposed as an alternative treatment option for X-ALD [14], [12], [15], [16]. After *ex vivo* gene therapy, the patients' own ABCD1-corrected CD34^+^-derived immune cells were able to halt the inflammation in CALD [9]. Thus, we recently investigated the VLCFA metabolism in the various CD34^+^-derived cell types of X-ALD patients and demonstrated that predominantly monocytes and granulocytes, but not lymphocytes, are metabolically affected by ABCD1 deficiency [17]. Furthermore, in the cell types that are severely affected, ABCD1 mRNA is highly expressed whereas ABCD2 is virtually absent. This indicates that T cells and B cells have no severe intrinsic defect, although probably being involved in the pathomechanism of X-ALD (recently reviewed in [6]). Thus, up-regulation of ABCD2 in monocytes/macrophages could be important for X-ALD therapy. We hypothesize that the curative action of HSCT relies primarily on the exchange of the monocyte/macrophage-lineage. Thus, our overall goal is to pharmacologically induce ABCD2 in monocytes/macrophages, in order to rapidly stop the inflammation in patients with unfavorable progression for HSCT.

**Figure 1:**
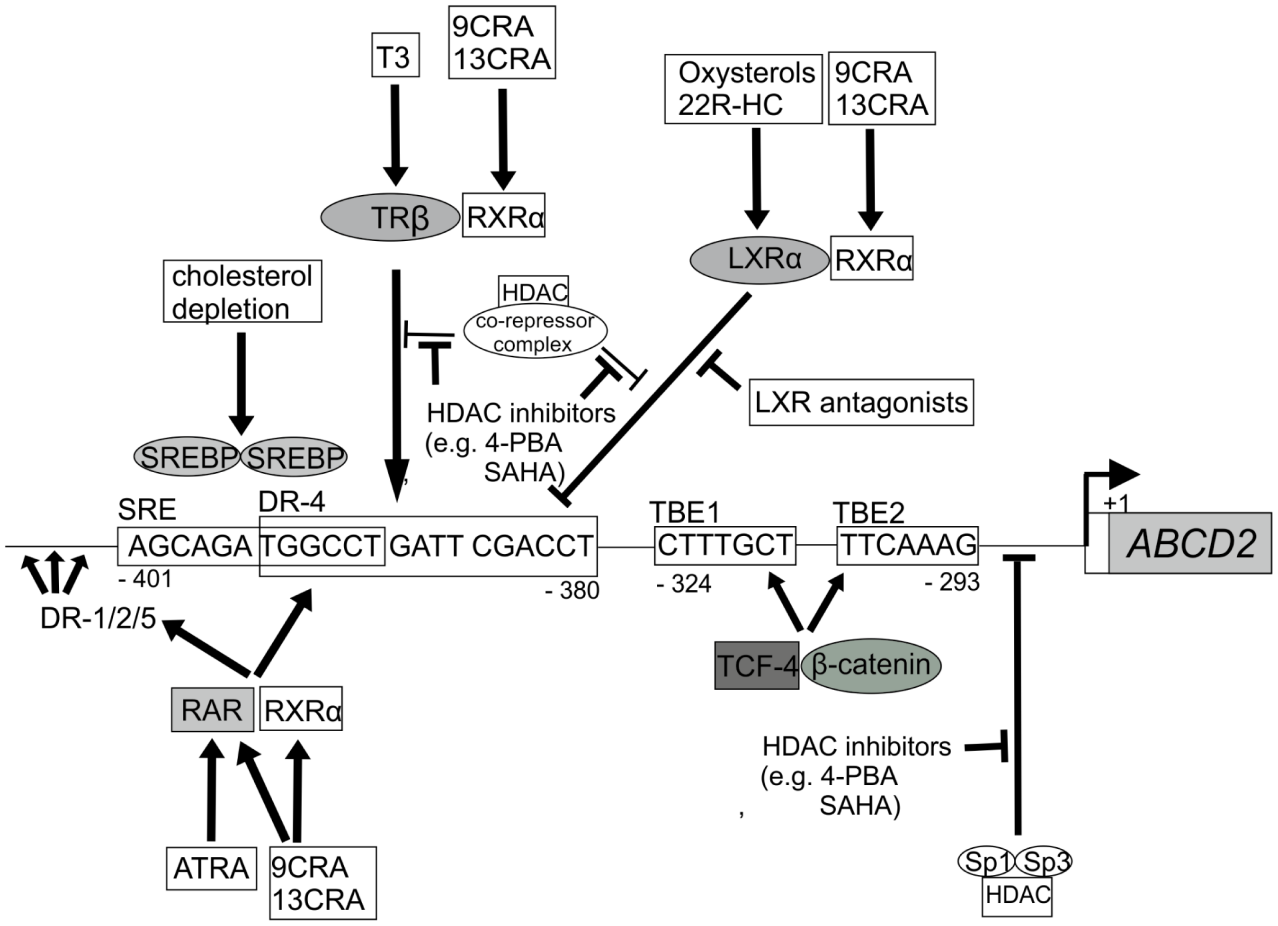
A complex regulatory network operates at the human *ABCD2* promoter. Activation and inhibition of the *ABCD2* gene are indicated. Nucleotide positions relative to the translational start site are indicated below the sequence. SREBP = sterol regulatory element (SRE)-binding proteins; LXR = liver X receptor; TR = thyroid hormone receptor; RXR = retinoid X receptor; RAR = retinoic acid receptor; TCF-4 = T-cell factor 4; Sp-1/3 = specificity protein 1/3; DR = direct repeat spaced by 1/2/4 or 5 nucleotides; TBE 1/3 = TCF binding element; 22R-HC = 22(R)-hydroxycholesterol; 9/13CRA = 9/13-*cis*-retinoic acid; T3 = triiodothyronine; 4-PBA = 4-phenylbutyrate; SAHA = suberoylanilide hydroxamic acid.

Promoter analyses of the *ABCD2* gene have revealed a complex network of transcription factors (Fig. 1) that sense the intracellular levels of steroid hormones and lipids including sterol regulatory element (SRE)-binding proteins (SREBPs), as well as nuclear receptors like the liver X receptor (LXR), thyroid hormone receptor (TR) and retinoid X receptor (RXR) [18]–[22]. In addition, the *ABCD2* gene can be up-regulated by inhibitors of histone deacetylases (HDAC) like 4-phenylbutyrate and suberoylanilide hydroxamic acid (SAHA) [14], [23], indicating that also epigenetic mechanisms play a role in *ABCD2* expression. Recently, also the T-cell factor 4 (TCF-4) and β-catenin, both of which are factors in the Wnt-signaling pathway regulating renewal and differentiation of hematopoietic stem cells, were shown to participate in *ABCD2* regulation [24].

Retinoic acid (RA), a bioactive derivative of vitamin A, is able to induce *ABCD2* expression in cultured cells [25], [15]. RA exists in two different isomeric forms, all-*trans*-retinoic acid (ATRA) and 9-*cis*-RA (9CRA), and acts by binding to the nuclear retinoic acid receptor (RAR) and retinoid X receptor (RXR) [26]. These receptors directly act as transcription factors (Fig. 1). Intriguingly, RXR also forms heterodimers with other nuclear receptors known to regulate *ABCD2* expression, as for example LXR and TR; thus, RA is emerging as a key player in *ABCD2* regulation (Fig. 1). Moreover, RA signaling is directly linked to development and effector functions of various immune cell types including monocytes and macrophages [27]. In addition, retinoids exhibit anti-inflammatory action by inhibiting various immune responses including activity of leukocytes and release of proinflammatory cytokines [28]. Taken together, retinoids are attractive compounds in the context of X-ALD. Thus, the aim of this study was to investigate the capacity of retinoids to induce *ABCD2* expression in human monocytes *in vivo* and in primary *in vitro* differentiated macrophages.

## Results

### Retinoids induce ABCD2 mRNA levels in human macrophage THP-1 cells

**Figure 2:**
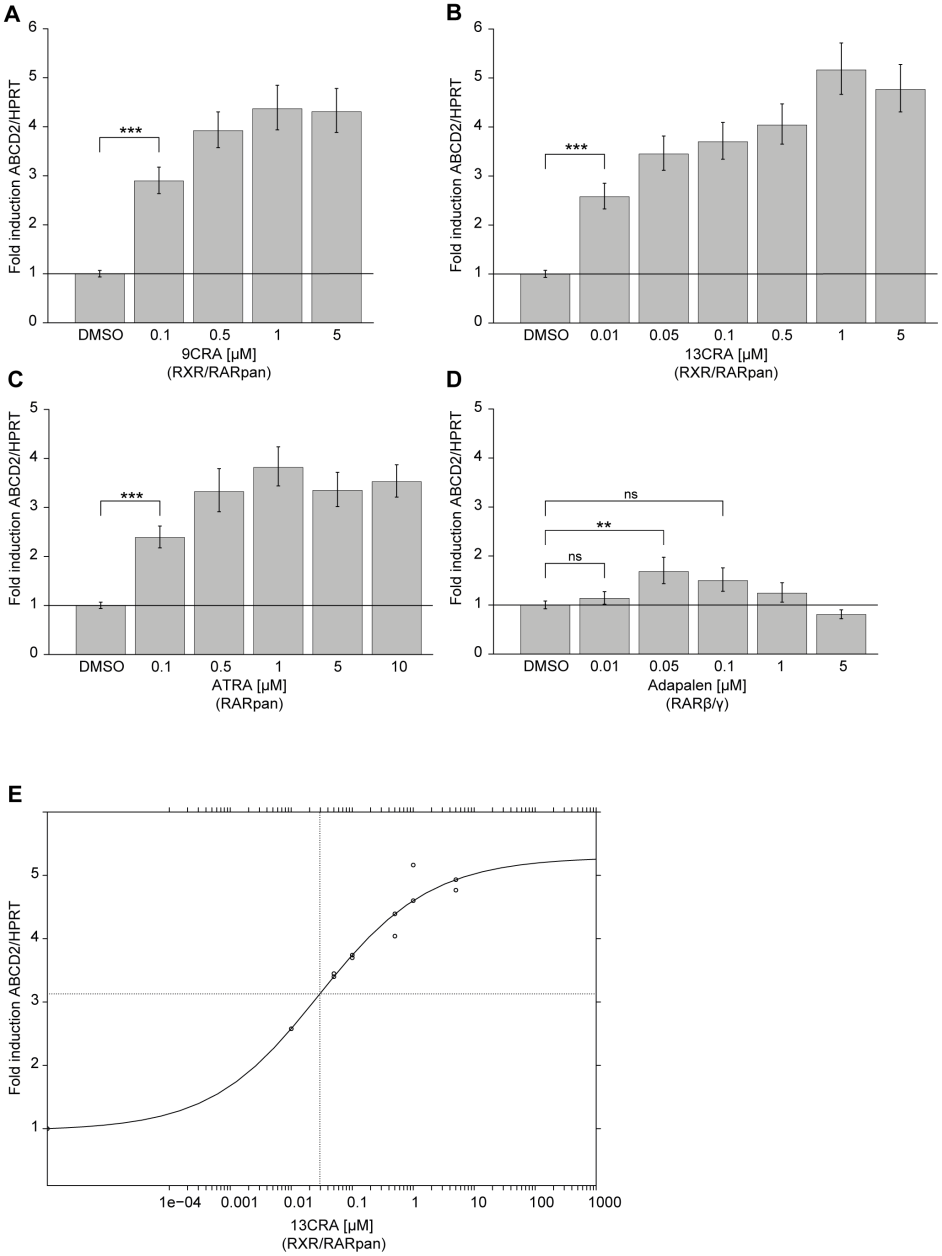
Agonists of RAR and RXR induce ABCD2 mRNA expression in THP-1 cells. The mRNA levels of ABCD2 and the reference gene HPRT were determined by qRT-PCR after 24-h-treatment with the indicated concentrations of (A) 9CRA, (B) 13CRA, (C) ATRA and (D) Adapalene. The receptor-selectivity of each ligand is indicated below the panels. (E) Dose-response curve for 13CRA. Values represent means ± SEM of treated cells as fold-induction relative to solvent (DMSO)-treated cells. Each column represents three independent treatments. qRT-PCR analyses were performed in 2–3 technical replicates. ** p<0.01; *** p<0.001; *ns*, not significant.

To evaluate the potential of retinoids for induction of *ABCD2* expression in a monocyte/macrophage lineage, we first used the human THP-1 monocyte-like suspension cell line. Cells were treated either with the natural pan RAR and RXR agonists 9CRA (Fig. 2A) or 13-*cis*-RA (13CRA) (Fig. 2B) or with the RAR-selective agonist ATRA (Fig. 2C). In addition, we tested the synthetic derivative Adapalene, which specifically activates the RAR β and γ isoforms (Fig. 2D). Cells were treated for 24 h with the indicated compounds and doses; thereafter ABCD2 mRNA levels were measured by quantitative reverse transcription-coupled PCR (qRT-PCR), using the reference gene hypoxanthine phosphoribosyltransferase (HPRT) for normalization. With Adapalene only a twofold (p<0.05) induction of ABCD2 mRNA relative to the solvent (DMSO) was observed (Fig. 2D). Treatment with the biologically active retinoic acids 9CRA, 13CRA and ATRA showed a comparable (3.5 to 5-fold), statistically significant (p<0.001) *ABCD2* induction (Fig. 2A, B and C). Differences existed in the concentration of the different retinoids required to achieve the maximal induction of *ABCD2* expression. With a fivefold induction, 13CRA generated the highest level of ABCD2 mRNA and showed a dose dependent effect with a half maximal effective concentration (EC_50_) of 0.03 µM (Fig. 2E). ATRA and 9CRA required higher concentrations (1 µM) for a similar level of induction. A smaller response was obtained with several other retinoids (data not shown). Thus, of these compounds, 13CRA was the most effective at inducing *ABCD2* expression in the THP-1 cell line and we decided to evaluate this retinoid *in vivo*.

### Evaluation of the ability of 13CRA to induce ABCD2 mRNA in human primary monocytes *in vivo*

**Figure 3:**
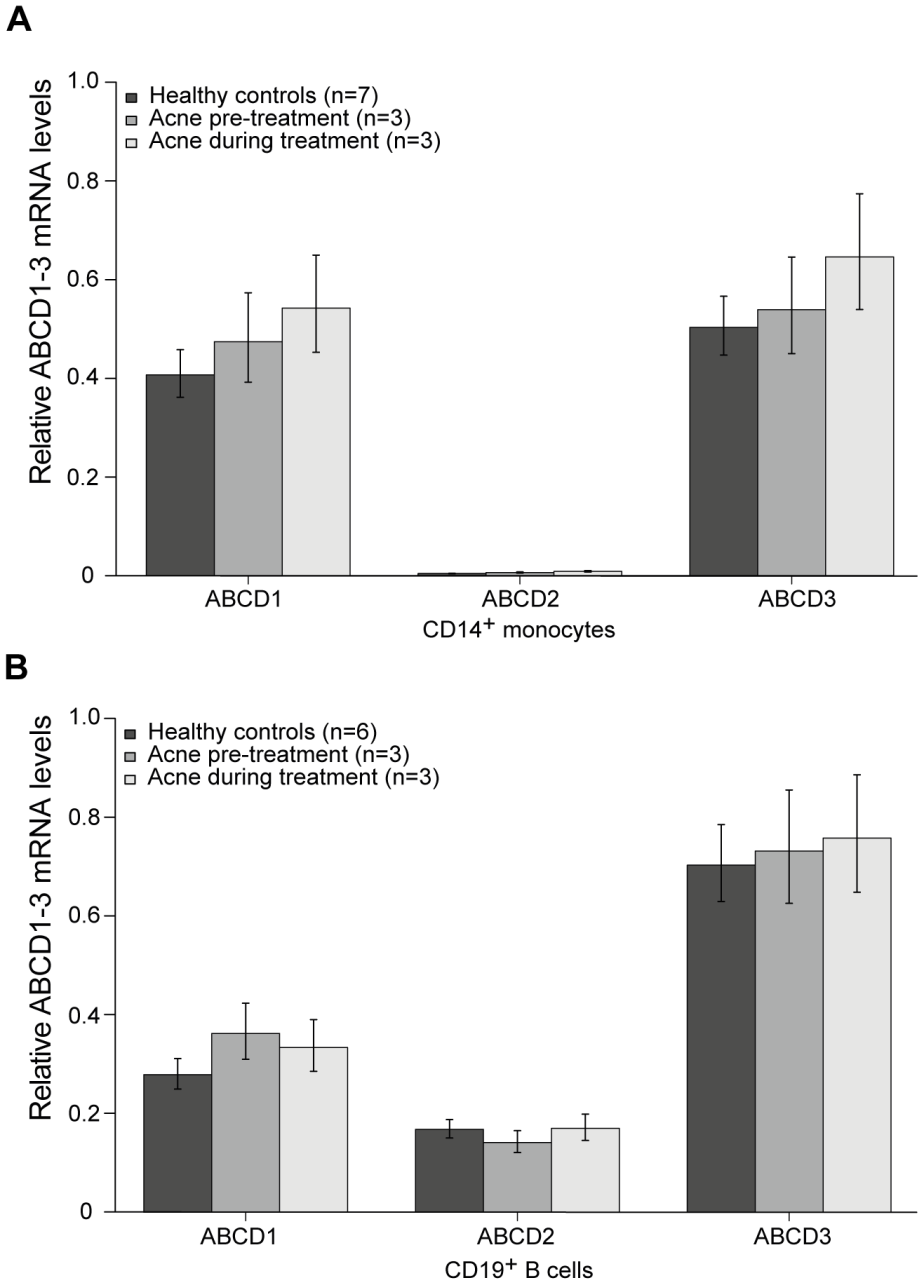
ABCD2 mRNA is not induced in monocytes or lymphocytes of acne patients treated with 13CRA. ABCD1, ABCD2, ABCD3 and HPRT mRNA levels were measured by qRT-PCR in (A) monocytes (CD14^+^) and (B) B cells (CD19^+^) of healthy controls and acne patients before and after oral treatment with 13CRA for a period of about 3 months. HPRT was used for normalization of the absolute mRNA copy numbers. Values represent means ± SEM; qRT-PCR analyses were performed in technical duplicates. The number of individuals (*n*) is indicated in the inserts.

Retinoids, in particular 13CRA is in clinical use as a standard medication (Isotretinoin) to treat severe acne. The male patient collective received a daily oral dose of 0.75 mg/kg body weight (or a cumulative dose of 120 mg/kg) for an initial period of approximately six months, with the option of further extension. After ethical approval, blood samples were taken in the morning under fasting conditions before and during the treatment time. All patients included in our analysis showed a successful reduction of their cystic acne under the treatment regime. We analyzed the effect of 13CRA on the mRNA levels of the peroxisomal ABC transporters (ABCD1, ABCD2, ABCD3) and HPRT (for normalization) in isolated CD14^+^ monocytes, CD19^+^ B cells and the T cell-enriched PBMC fraction remaining after depletion of the CD14^+^ and CD19^+^ cells (approximately 75% T cells) of acne patients receiving 13CRA therapy. Before starting the treatment, the mRNA levels of all three peroxisomal ABC transporters were comparable to those of healthy controls in monocytes and B cells (Fig. 3A, B). However, in contrast to our results from THP-1 cells, 13CRA did not alter ABCD2 mRNA levels in any of the investigated cell types after a treatment period of about three months. Also the ABCD1 and ABCD3 mRNAs remained at pre-treatment levels (Fig. 3 A, B). The T cell-enriched peripheral blood mononuclear cells (PBMC) fraction after depletion of monocytes and B cells did also not show any alterations before and during the treatment with 13CRA (see Fig. S1). In two of the three acne patients additional measurements were performed after six months and for one patient after nine months of 13CRA treatment with identical results as for the three months time point (see Fig. S2).

### Treatment of human primary monocyte-derived macrophages with 13CRA

**Figure 4:**
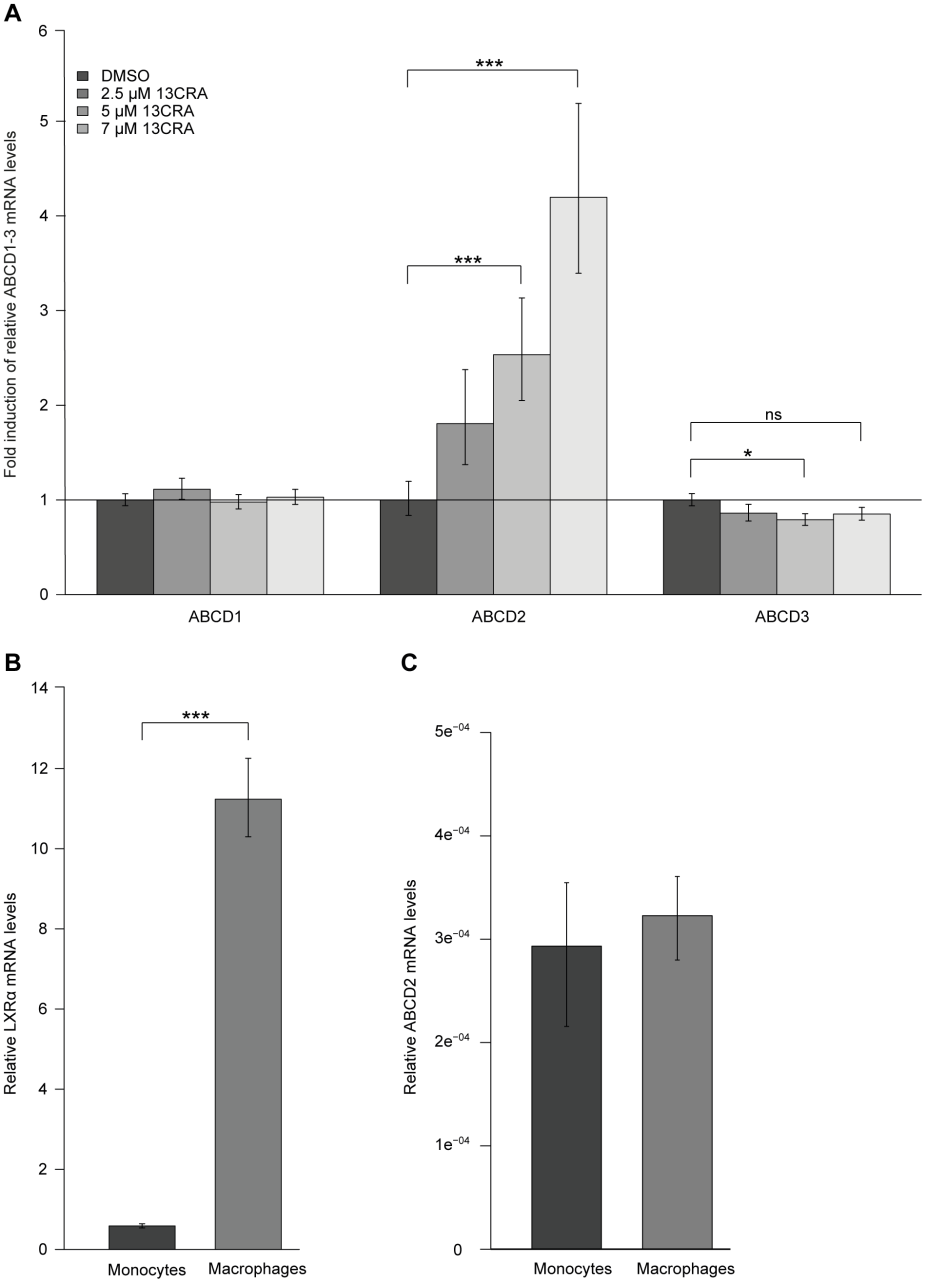
13CRA mediates a modest induction of *ABCD2* in differentiated human macrophages *in vitro*. (A) ABCD1, ABCD2, ABCD3, and, as a reference for normalization, HPRT mRNA levels were detected by qRT-PCR after treatment of *in vitro* differentiated macrophages with 2.5, 5 and 7 µM of 13CRA for 24 h. The results are shown as fold-induction of ABCD1-3/HPRT relative to solvent (DMSO)-treated cells. (B) As a control for the differentiation of monocytes into macrophages, LXRα expression was quantified and depicted as copy numbers of LXRα mRNA normalized to HPRT mRNA levels. (C) ABCD2 mRNA levels were similar in monocytes and M-CSF-differentiated macrophages. Values represent means ± SEM of three independent treatments. qRT-PCR analyses were performed in two technical replicates. * *p*<0.05; ** *p*<0.01; *** *p*<0.001; *ns*, not significant.

Since monocytes are precursor cells of macrophages and these appear to be strongly affected by X-ALD pathology, we next tested if 13CRA is able to induce *ABCD2* expression in human monocyte-derived macrophages. To this end, CD14^+^ monocytes were isolated from the blood of healthy untreated donors and differentiated *in vitro* with macrophage-colony stimulating factor (M-CSF) for 7 days. These monocyte-derived macrophages were incubated with different concentrations of 13CRA (2.5, 5 and 7 µM) for 24 h, thereafter RNA was isolated and ABCD1, ABCD2 and ABCD3 mRNA levels were quantified by qRT-PCR and normalized to HPRT (Fig. 4). LXRα expression was monitored as a control for macrophage differentiation [29]. Treatment of monocyte-derived macrophages with 13CRA resulted in a dose-dependent induction of *ABCD2* expression, with the highest increase (fourfold) observed at a concentration of 7 µM (Fig. 4A). ABCD1 mRNA levels remained unchanged, whereas *ABCD3* expression decreased slightly starting with the lowest concentration 2.5 µM 13CRA. As expected, LXRα was highly induced (approximately 14-fold) in macrophages compared with unstimulated monocytes, indicating an appropriate differentiation of monocytes into macrophages in response to M-CSF (Fig. 4B). ABCD2 mRNA levels were not affected by the differentiation and remained at the same low level in both populations (Fig. 4C).

## Discussion

It is an urgent need to develop treatment strategies to halt the devastating inflammation in CALD patients in general, but especially in patients that cannot be considered for HSCT and during the time window until transplanted cells are able to stop the inflammation. For these cases, pharmacological induction of the redundant gene *ABCD2*, in the monocyte/macrophage lineage could be an alternative or additional treatment option. Extensive promoter analyses and former experiments suggested retinoids as promising candidate drugs for X-ALD [15], [21], [30]. Here, we tested the ability of retinoids to induce *ABCD2* expression in human primary monocytes. This cell type is especially vulnerable to ABCD1 deficiency, because virtually no ABCD2 mRNA is present, possibly due to epigenetic silencing of the gene in these cells [17]. Of the various tested retinoids, 13CRA achieved the highest relative induction of *ABCD2* expression in THP-1 cells and thus, was further tested *in vivo*. However, in human primary monocytes isolated from acne patients treated with 13CRA, ABCD2 mRNA levels were unchanged when compared with pretreatment levels or healthy controls. This finding may indicate that retinoids alone are not able to revert the silencing/repression of *ABCD2* expression in human primary monocytes.

As monocytes are the direct precursor cells of macrophages, and macrophages were shown to have an important role in X-ALD pathology by invading active brain lesions [31], we also tested whether 13CRA is able to stimulate *ABCD2* expression in monocyte-derived *in vitro* differentiated macrophages. Indeed, 13CRA treatment resulted in a fourfold induction of *ABCD2* expression. However, in the context of X-ALD, where ABCD2 is needed as a substitute for ABCD1 deficiency, a fourfold increase in ABCD2 mRNA levels is still low when compared with the relatively high levels of ABCD1 present in healthy monocytes. Thus, future experiments are necessary to a) evaluate the therapeutic relevance of a fourfold induction of *ABCD2* expression in human macrophages and b) to identify drugs that confer higher levels of ABCD2 induction, possibly by combining retinoids with epigenetic compounds acting to reverse silencing of ABCD2 expression in the monocyte/macrophage lineage.

In summary, we conclude that retinoids are probably not able to induce *ABCD2* to a level high enough for a compensation of ABCD1 deficiency in macrophages *in vivo*. It will be a major challenge for a pharmacological induction of *ABCD2* gene expression in monocytes/macrophages, or in microglia, to overcome the silenced status of *ABCD2* in these cell types.

## Materials and Methods

### Nomenclature

Throughout the manuscript we have used the nomenclature for nuclear receptors and agonists according to the agreed nomenclature by the NC-IUPHAR Committee of Nuclear Receptors [32], [33].

### Patients & healthy controls

Included in our analyses were three acne patients (mean age in years 18.3±2.9) and seven healthy controls (mean age in years 30±7.7) of European origin. We recruited acne patients older than 14 years, who required treatment with Isotretinoin because of the severity of their disease. For the healthy volunteers, we included male subjects older than 18 years, healthy at the time of blood sampling and not taking any medication. During a typical six-month-treatment with retinoic acid, there are regularly scheduled blood controls (before and during the treatment, approximately every three months), at which an additional blood sample was obtained for our study. From patients and healthy controls, 30 ml of venous blood was collected in the morning under fasting conditions. As two of the acne patients were below the age of 18 years, the parents signed an additional written informed consent on behalf of the children. All volunteers were enrolled in accordance to the Declaration of Helsinki and signed written informed consent. The study was approved by the Ethical Committee of the Medical University of Vienna (EK No. 042/2011; 437/2010).

### Treatment of patients

Acne patients received 13CRA (Isotretinoin, Roaccutane, Roche Ltd., Mississauga, Canada) at a daily oral dose of 0.75 mg/kg body weight (cumulative dose 120 mg/kg) for an initial period of six months.

### Cell purification and evaluation of purity

Venous blood was collected into heparin tubes, diluted 1∶1 with phosphate-buffered saline (PBS), layered onto tubes filled with 15 ml of Pancoll separating medium (density, 1.077 g/ml; PAN-Biotech, Aidenbach, Germany) and centrifuged for 25 min at 500×*g* without brake at room temperature. PBMC were retrieved and washed with PBS. Monocytes (CD14^+^) and B cells (CD19^+^) were isolated from the PBMC by immunomagnetic (MACS) positive selection, according to the instructions of the manufacturer (Miltenyi Biotec, Bergisch Gladbach, Germany). The following microbead-coupled antibodies were used: CD14^+^ (Product No. 130-050-201) and CD19^+^ (Product No. 130-050-301). The PBMC fraction remaining after depletion of CD14^+^ and CD19^+^ cells was also retrieved and considered “T cell-enriched PBMC” (approximately 75% T cells). Cell purity was assessed on a BD FACS Calibur flow cytometer (Becton Dickinson, Franklin Lakes, NJ, USA) and evaluated with FlowJo software (Treestar Inc., Ashland, OR, USA).

### *In vitro* differentiation of primary human monocytes to macrophages

CD14^+^ monocytes isolated from healthy human donors as described above were differentiated *in vitro* to a mature macrophage phenotype by incubating cells in RPMI-1640 medium including 2 mM L-glutamine (PAA, Pasching, Austria) supplemented with 10% heat-inactivated fetal bovine serum (Life Technologies, Paisley, UK), 100 µg/ml streptomycin (Lonza), 100 units/ml penicillin (Lonza) and 1 µg/ml Fungizone (Invitrogen, Paisley, UK) in the presence of 50 ng/ml M-CSF (PeproTech, Rocky Hill, NJ, USA) for 7 days.

### Cell line

THP-1, a human monocyte/macrophage-like suspension cell line was obtained from American Type Culture Collection (Manessas). THP-1 cells were seeded at a density of 2×10^5^ cells/well in 6-well plates (Greiner bio-one, San Diego, US) in 2.5 ml RPMI 1640 medium including 2 mM L-glutamine (PAA) supplemented with 10% heat-inactivated fetal bovine serum (PAA), 100 µg/ml streptomycin (Lonza), 100 units/ml penicillin (Lonza) and 1 µg/ml Fungizone (Invitrogen) in a 5% CO_2_ atmosphere at 37°C.

### *In vitro* drug treatment

The following drugs were used for *in vitro* experiments in the THP-1 cell line: 9-*cis*-retinoic acid (9CRA 0.1, 0.5, 1 and 5 µM; Sigma, St. Louis, US); 13-*cis*-retinoic acid (13CRA 0.01, 0.05, 0.1, 0.5, 1 and 5 µM; Sigma); All-*trans*-retinoic acid (ATRA 0.5, 1, 5 and 10 µM; Sigma) and naphthenic acid derivate (Adapalene 0.01, 0.05, 0.1 µM; Tocris Bioscience, Ellisville, US). For drug treatment of *in vitro* differentiated macrophages (8×10^5^ cells/well in 12-well plates) 13CRA was added at: 2.5, 5 and 7 µM final concentrations. Chemical compounds were dissolved in DMSO (Sigma) as 10 mM stock solutions for ATRA, 9CRA and 13CRA; and 1 mM for Adapalene and stored at −80°C. The compounds were further diluted in DMSO such that the final concentration of DMSO in the medium was always 0.5% (vol/vol). Treatments lasted for 24 h and were performed in triplicates.

### Quantitative reverse transcription-coupled PCR analysis (qRT-PCR)

Cells were lysed in TRIzol reagent (Invitrogen) and total RNA was isolated following manufacturer's instructions. After centrifugation through a QIAshredder spin column (Qiagen, Bothell, WA), the RNA fraction was further purified using silica membrane-based columns (RNeasy Mini Kit) and RNase-free DNase digestion (both from Qiagen). RNA concentrations were measured based on optical density using a Nanodrop spectrophotometer (Peqlab). Total RNA was reverse transcribed using the iScript cDNA Synthesis Kit (BioRad, Hercules, CA) and the diluted cDNA was amplified in two or three technical replicates by qRT-PCR as previously described [17]. Briefly, ABCD1, ABCD3, LXRα and HPRT were detected by the “SYBRGreen” method using SsoFast EvaGreen Mix (BioRad). ABCD2 was detected by the Taqman method using SsoFast Probes Supermix (BioRad). Both methods were carried out using the CFX96 Realtime System (BioRad) according to the manufacturer's instructions. For amplification and detection of ABCD1 cDNA the primers 5′-GAGAACATCCCCATCGTC-3′ (forward, nucleotide position 1828) and 5′-TGTAGAGCACACCACCGTA-3′ (reverse, nucleotide position 1996) were used (GenBank Accession No. NM_000033.3). For ABCD2 cDNA the primers 5′-TCCTACACAATGTCCATCTCT-3′ (forward, nucleotide position 1883) and 5′-AGGACATCTTTCCAGTCCA-3′ (reverse, nucleotide position 1961) as well as the TaqMan fluorescent probe 5′-Cy5-CAAAGAGAAGGAGGATGGGATGC-BHQ2-3′ (nucleotide position 1915) were used (GenBank Accession No. AJ000327.1). For ABCD3 the primers 5′-CGGCTCATCACAAACAGTGA-3′ (forward, nucleotide position 811) and 5′-AGGTGTTCCACCAGTTTTCG-3′ (reverse, nucleotide position 908) were used (GenBank Accession No. M81182.1). For LXRα, the primers 5′-CAGGGCTGCAAGTGGAATTCA-3′ (forward, nucleotide position 1027) and 5′-TCCGGAGGCTCACCAGTTTC-3′ (reverse, nucleotide position 1287) were used (GenBank Accession No. NM_001130101.1). For HPRT the primers 5′-CCCTGGCGTCGTGATTAGT-3′ (forward, nucleotide position 182) and 5′-CAGGTCAGCAAAGAATTTATAGCC-3′ (reverse, nucleotide position 401) were used (GenBank Accession No. NM_000194).

### Statistical Analyses

We used R [34] and the nonlinear mixed effect models (nlme) package [35]–[37] to perform a linear mixed effects analysis of the relationship between ABCD1-3 mRNA expression levels and retinoid treatments in THP-1 cell line; monocytes and B cells of acne patients and healthy controls; T cell-enriched PBMC of acne patients and in differentiated macrophages. As fixed effects, we entered GeneType (primary Covariate: HPRT, ABCD1-3) and DrugTreatment with an interaction term into the model. As a grouping factor, we used subject for *in vivo* study and sample for *in vitro* experiments. As a random effect model, we had intercepts and by-group slopes for the effect of GeneType. A variance stabilizing log-transformation of the response (mRNA ABCD1-3 transporter expression levels) was used for all analyses. Visual assessment of residual plots did not show any deviations from normality or homoscedasticity. We tested the overall significance of terms in the fixed effect model using the conditional F test. The levels of a significant fixed effect term were further tested using Students *t* tests. Multiple testing was taken into account using *p*-values from a multivariate T-distribution [38].

Full linear mixed model formula:
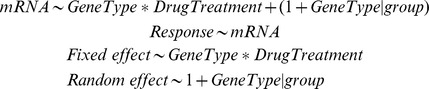



## Supporting Information

Figure S1 **ABCD2 mRNA is not induced in T cell-enriched PBMC of acne patients treated with 13CRA.** ABCD1, ABCD2, ABCD3 and HPRT mRNA levels were measured by qRT-PCR in the T cell-enriched PBMC of three acne patients before and after oral treatment with 13CRA for a period of about 3 months. HPRT was used for normalization of the absolute mRNA copy numbers. Values represent means ± SEM; qRT-PCR analyses were performed in technical duplicates.
(TIF)

Figure S2 **Time course of ABCD2 mRNA levels in monocytes of acne patients treated with 13CRA.** Pre-treatment values are indicated as time point 0. For patient 2 and 3, there are additional measurements after 6 months (around 200 days) and for patient 2 after 9 months (300 days) and after 15 months (450 days), about 3 months post-treatment with 13CRA. ABCD2 mRNA is not induced in monocytes of acne patients treated with 13CRA.
(TIF)
