# Supplementary figures and images for: Evaluation of Retinoids for Induction of the Redundant Gene ABCD2 as an Alternative Treatment Option in X-Linked Adrenoleukodystrophy
Source: PLoS One. 2014 Jul 31;9(7):e103742. doi: 10.1371/journal.pone.0103742 (PMC4117577; doi:10.1371/journal.pone.0103742)

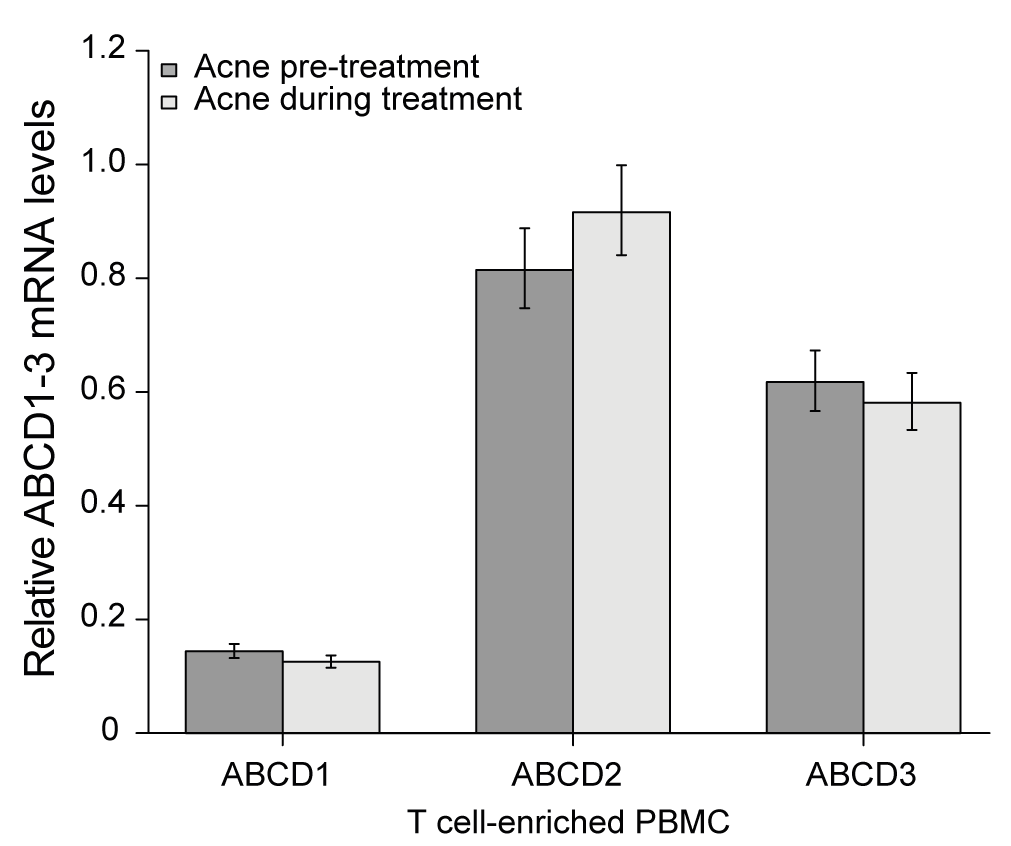

Supplement: Figure S1 — ABCD2 mRNA is not induced in T cell-enriched PBMC of acne patients treated with 13CRA. ABCD1, ABCD2, ABCD3 and HPRT mRNA levels were measured by qRT-PCR in the T cell-enriched PBMC of three acne patients before and after oral treatment with 13CRA for a period of about 3 months. HPRT was used for normalization of the absolute mRNA copy numbers. Values represent means ± SEM; qRT-PCR analyses were performed in technical duplicates. (TIF) [file pone.0103742.s001.tif]

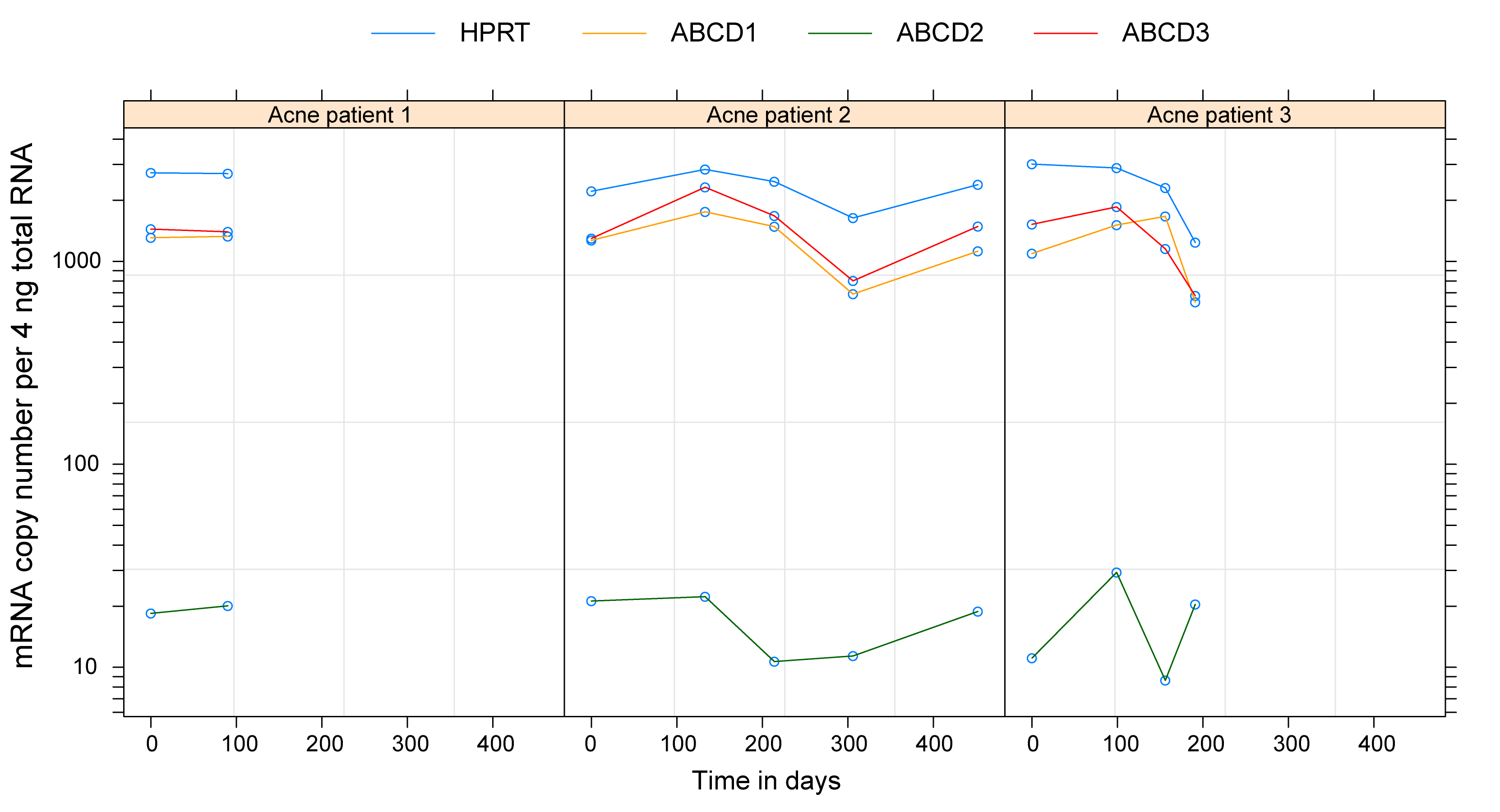

Supplement: Figure S2 — Time course of ABCD2 mRNA levels in monocytes of acne patients treated with 13CRA. Pre-treatment values are indicated as time point 0. For patient 2 and 3, there are additional measurements after 6 months (around 200 days) and for patient 2 after 9 months (300 days) and after 15 months (450 days), about 3 months post-treatment with 13CRA. ABCD2 mRNA is not induced in monocytes of acne patients treated with 13CRA. (TIF) [file pone.0103742.s002.tif]
